# Dense, single-phase, hard, and stress-free Ti_0.32_Al_0.63_W_0.05_N films grown by magnetron sputtering with dramatically reduced energy consumption

**DOI:** 10.1038/s41598-022-05975-5

**Published:** 2022-02-09

**Authors:** X. Li, B. Bakhit, M. P. Johansson Jõesaar, I. Petrov, L. Hultman, G. Greczynski

**Affiliations:** 1 Thin Film Physics Division, Department of Physics (IFM), Linköping University, SE-581 83 Linköping, Sweden; 2 SECO Tools AB, SE-737 82 Fagersta, Sweden; 3 Materials Research Laboratory, University of Illinois, Urbana, IL 61801 USA; 4 Department of Materials Science and Engineering, National Taiwan University of Science and Technology, Taipei, 10607 Taiwan

**Keywords:** Surfaces, interfaces and thin films, Mechanical properties, Ceramics

## Abstract

The quest for lowering energy consumption during thin film growth, as by magnetron sputtering, becomes of particular importance in view of sustainable development goals. A recently proposed solution combining high power impulse and direct current magnetron sputtering (HiPIMS/DCMS) relies on the use of heavy metal-ion irradiation, instead of conventionally employed resistive heating, to provide sufficient adatom mobility, in order to obtain high-quality dense films. The major fraction of process energy is used at the sputtering sources rather than for heating the entire vacuum vessel. The present study aims to investigate the W^+^ densification effects as a function of increasing Al content in (Ti_1-*y*_Al_*y*_)_1-*x*_W_*x*_N films covering the entire range up to the practical solubility limits (*y* ~ 0.67). Layers with high Al content are attractive to industrial applications as the high temperature oxidation resistance increases with increasing Al concentration. The challenge is, however, to avoid precipitation of the hexagonal wurtzite AlN phase, which is softer. We report here that (Ti_1-*y*_Al_*y*_)_1-*x*_W_*x*_N layers with *y* = 0.66 and *x* = 0.05 grown by a combination of W-HiPIMS and TiAl-DCMS with the substrate bias *V*_*s*_ synchronized to the W^+^-rich fluxes (to provide mobility in the absence of substrate heating) possess single-phase NaCl-structure, as confirmed by XRD and SAED patterns. The evidence provided by XTEM images and the residual oxygen content obtained from ERDA analyses reveals that the alloy films are dense without discernable porosity. The nanoindentation hardness is comparable to that of TiAlN films grown at 400–500 °C, while the residual stresses are very low. We established that the adatom mobility due to the heavy ion W^+^ irradiation (in place of resistive heating) enables the growth of high-quality coatings at substrate temperatures not exceeding 130 °C provided that the W^+^ momentum transfer per deposited metal atom is sufficiently high. The benefit of this novel film growth approach is not only the reduction of the process energy consumption by 83%, but also the possibility to coat temperature-sensitive substrates.

## Introduction

Physical vapor deposition (PVD) techniques are an essential ingredient of modern materials processing with numerous application areas, e.g., hard coatings^1,2^, optical films^3^, films for microelectronic circuits^4^, transparent conductive oxides^5^, thin film solar cells^6^, and will continue to do so in the decades to come. It is, thus, essential to make them energy-efficient to comply with current sustainable development goals. Thin film growth by PVD methods consumes high amounts of energy primarily for substrate heating to provide sufficient adatom mobility and ensure that dense layers are obtained. Films grown with no external heating are typically underdense, which results in poor properties^7^. Another aspect is that high deposition temperatures, typically 400–600 °C, prevent depositions on heat-sensitive substrates, thus effectively excluding modern engineering materials such as lightweight Al- and Mg-based alloys.

A possible solution demonstrated in 2014 relies on the use of a hybrid high-power impulse and dc magnetron co-sputtering (HiPIMS/DCMS) involving a high mass (*m*_*Me*_ > 180 amu) HiPIMS target and metal-ion-synchronized bias pulses^8,9^. To maintain a high deposition rate, the primary metal targets operate in DCMS regime^10^, while a high-mass target powered by HiPIMS supplies energetic metal-ion flux irradiation which densifies the deposited material due to the effective low-energy recoil generation resulting from the large mass mismatch between the incident ion and film constituents^11^. Thus, the deposition rate is high, while the deposition temperature *T*_*s*_ can be kept low as no intentional substrate heating is supplied. The energy efficiency of the PVD process is increased tremendously since majority of the supplied energy is consumed by the sputtering cathodes rather than being wasted for heating the entire vacuum vessel as is the case in conventional processing. The heavy metal-ion energy and momentum transfer to the growing film surface is controlled by the amplitude of the substrate bias *V*_*s*_, which is synchronized to the metal-ion-rich portions of the HiPIMS plasma using the input from ion mass spectrometry analyses performed at the substrate position^12–14^. During the DCMS phase (i.e., between HiPIMS pulses), the substrate is intentionally kept at floating potential to minimize the role of gas ions that arrive with energies lower than the lattice displacement threshold (~ 20–50 eV, depending upon the ion and film species involved), i.e., too low to result in efficient gas entrapment and high compressive stress levels^15,16^.

Early examples of advantages offered by a hybrid HiPIMS/DCMS technique with metal-ion-synchronized substrate bias include the use of Ta^+^ (*m*_*Ta*_ = 181.0 amu) or W^+^ HiPIMS ions (*m*_*W*_ = 183.8 amu) to grow dense and hard Ti_1-*x-y*_Al_*x*_Ta_*y*_N^9^, Ti_1-*x*_Ta_*x*_N^8^, or Ti_0.40_Al_0.27_W_0.33_N^17^ films at the substrate temperatures not exceeding 150 °C. The high quality of these layers was confirmed by the application studies in which Ti_1-*x*_Ta_*x*_N layers performed exceptionally well as Cu diffusion barriers on Si (001)^18^ and provided excellent corrosion protection for stainless-steel substrates^19^. Systematic studies of densification effects induced by group VIB transition metal (TM) target ions in Ti_0.50_Al_0.50_ N demonstrated that the densification effects scale with the mass of HiPIMS-generated metal-ions^20^. (Ti_1-*y*_Al_*y*_)_1-*x*_W_*x*_N films were dense even with the lowest W concentration, *x* = 0.09, showed no evidence of hexagonal wurtzite AlN precipitation, and exhibit state-of the-art mechanical properties typical of Ti_0.50_Al_0.50_ N grown at 500 °C.

In the follow-up study that focused specifically on the effects of the W^+^ irradiation two essential parameters were varied: W^+^ energy ${E}_{{W}^{+}}$ (from ~ 90 to ~ 630 eV) and W concentration on the metal lattice *x* = W/(W + Ti + Al) (0.02 ≤ *x* ≤ 0.12)^21^. The former was controlled with the amplitude of the substrate bias pulses and the latter by varying HiPIMS pulse width, while keeping the peak target current and pulsing frequency constant. Results revealed a strong coupling between the W^+^ incident energy and the minimum W concentration required to grow dense layers. Importantly, for all tested combinations of ${E}_{{W}^{+}}$ and *x*, single phase NaCl structure films were obtained, indicating that energetic high-mass metal-ion irradiation of Ti_0.52_Al_0.48_N does not promote precipitation of softer hexagonal wurtzite AlN phase*.*

The present study aims to investigate the W^+^ densification effects as a function of increasing Al content in (Ti_1-*y*_Al_*y*_)_1-*x*_W_*x*_N films covering the entire range up to the practical solubility limits (*y* ~ 0.67). Previous experiments conducted with metal-ion-synchronized growth concept utilizing Al ions revealed that during high temperature growth, Ti_1-*y*_Al_*y*_N layers with *y* as high as 0.65 still maintain single-phase NaCl cubic structure^11,13,22^, Layers with high Al content are attractive to industrial applications as the high temperature oxidation resistance increases with increasing Al concentration. The challenge is, however, to avoid precipitation of the hexagonal wurtzite AlN phase, which is softer. The essential research question addressed in this contribution is then—what are the Al solubility limits if the adatom mobility is provided by heavy ion W^+^ irradiation rather than by resistive heating? Is the novel film growth approach, proposed in our previous papers and aiming at significantly reduced energy consumption during PVD growth, competitive against state of the art high-temperature processing?

## Experimental

Depositions of (Ti_1-*y*_Al_*y*_)_1-*x*_W_*x*_N films are performed in an industrial CC800/9 magnetron sputtering system from CemeCon AG (Würselen, Germany) using a hybrid W-HiPIMS/TiAl-DCMS scheme, with a pulsed W^+^-flux provided by the W target operated in HiPIMS mode while Ti^+^- and Al^+^-fluxes are generated by two TiAl DC targets symmetrically placed on both sides of the W target^20,21^. TiAl DC targets are composed of Ti plates (Ti > 99 wt%) with 15 mm diameter Al plugs (Al > 99 wt%) distributed along the racetrack. The relative Al content in the films *y* = Al/(Al + Ti) is tuned by using sets of TiAl targets (8.8 × 50 cm^2^ size) with different number of plugs, as summarized in Table 1.

**Table 1:** The list of target configurations used to deposit (Ti_1-*y*_Al_*y*_)_1-*x*_W_*x*_N films with varying Al/(Al + Ti) ratio.

Group	TiAl DC targets configuration	*y* = Al/(Al + Ti) in Ti_1-*y*_Al_*y*_N reference samples
1	TiAl 48 + TiAl 48	0.48 ± 0.01
2	TiAl 48 GM* + TiAl 48 GM*	0.53 ± 0.01
3	TiAl 60 + TiAl 48 GM*	0.58 ± 0.01
4	TiAl 70 + TiAl 70	0.68 ± 0.01

*GM refers to the target with different arrangement of Al plugs along the racetrack resulting in higher Al content in the films for the same number of Al plugs.

1.5 × 1.5 cm^2^ Si (001) substrates sequentially cleaned in acetone and isopropanol alcohol are mounted in the center of a sample holder facing the W target, with 21° angle between substrates and both DCMS targets and an 18 cm target-to-substrate distance. A calibrated thermocouple placed near the center of the stationary substrates is used to measure the temperature during the entire process, as shown in the schematic diagram of the deposition system in Ref.^20^. Two resistance heaters placed symmetrically on the front and back sides of the chamber produce heat for degassing the chamber before deposition, so that the base pressure reaches 0.3 mPa (2.25 × 10^–6^ Torr). During the two-hour heating before deposition, 2 kW power is applied on each heater in the first hour (resulting in the substrate temperature of ~ 110 °C) and then the power on both heaters is switched off during the second hour so that the chamber cools down to ~ 65 °C before deposition starts. Due to the plasma heating during deposition, the substrate temperature monitored by the thermocouple rises to ~ 130 °C in the end of deposition. During film growth, the N_2_ and Ar flow are set constantly at 148 sccm and 360 sccm separately to maintain a total gas pressure *P*_*tot*_ of 0.4 Pa (3 mTorr).

Three series of W-HiPIMS/TiAl-DCMS films are made, with HiPIMS pulse length *τ*_*ON*_ of 30, 50, and 100 μs and DC power of either 10 kW (*τ*_*ON*_ = 30 and 50 μs) or 9.5 kW (for *τ*_*ON*_ = 100 μs). The HiPIMS frequency is 200 Hz, and a constant average power applied to the W target *P*_*HIP*_ = 1.0, 2.0, and 2.9 kW for the *τ*_*ON*_ = 30, 50, and 100 μs series (for the (100 μs, 240 V) batch, *P*_*HIP*_ = 2.8 kW) is set to keep the peak target current density *J*_*max*_ at ~ 0.80 A/cm^2^. A 200-μs long negative substrate bias pulse *V*_*S*_*(t)* with 30-μs offset is synchronized to the HiPIMS cathode^12,14^. With deposition time between 6–15 min, the film thickness is in the range of 1.3–2.0 μm.

Ti_0.36_Al_0.64_ N reference film is deposited by DCMS from two TiAl60 targets powered up to 10 kW each. During growth the substrate is maintained at a negative floating potential of *V*_*f*_ = − 10 V.

The time-of-flight elastic recoil detection analysis (ToF-ERDA) measurement operated in a tandem accelerator is utilized to determine the elemental compositions in the (Ti_1-*y*_Al_*y*_)_1-*x*_W_*x*_N films. The incident angle of 36 MeV ^127^I^8+^ probe beam is 67.5° with respect to the surface normal of sample, and the recoils are detected at 45°^23^. The cross-sectional scanning electron microscopy (XSEM) that determines thickness and cross-sectional morphology of the films is performed on a LEO 1550 instrument. A FEI Tecnai G2 instrument operated at 200 kV is used for cross-sectional transmission electron microscopy (XTEM) analysis. XTEM specimens are prepared by ion milling following fine mechanical polishing in a Gatan precision ion miller. During ion milling, Ar^+^ beam of 5.0 keV energy incidents at 3° angle from both the substrate side and film side with rotation. In the final stage of thinning, the beam energy is reduced to 2.0 keV. Phase structure is determined by *θ-2θ* X-ray diffraction (XRD) as a function of the sample tilt angle *ψ* (the angle between the sample normal and the scattering plane defined by the incoming and outgoing X-ray beams)^24^ on a Philips X’Pert MRD system with point-focus Cu K_α_ radiation source. X-ray Photoelectron Spectroscopy (XPS) depth profiles are obtained in a Kratos Analytical instrument, with a base pressure of 1.1 × 10^–9^ Torr (1.5 × 10^–7^ Pa), using monochromatic Al *K*_*a*_ radiation (*h*_*γ*_ = 1486.6 eV) with the X-ray anode operated at 150 W. Sputter-etching is done with 0.5 keV Ar^+^ ions incident at 70° with respect to the surface normal. The Ar^+^ ion beam is rastered over an area of 3 × 3 mm^2^ and XPS spectra are obtained from the center area (0.3 × 0.7 mm^2^) of the etched region. To evaluate the residual stress level of all (Ti_1-*y*_Al_*y*_)_1-*x*_W_*x*_N films, an X-ray diffraction substrate curvature method is performed on a Panalytical Empyrean XRD system and the rocking curve measurements of Si (400) reflections. The nanoindentation hardness *H* for all films is acquired through an Ultra-Micro Indentation System nanoindenter. The diamond probe used for indents is a sharp Berkovich type one. 30 indents are carried out on each sample.

## Results and discussion

**Figure 1:**
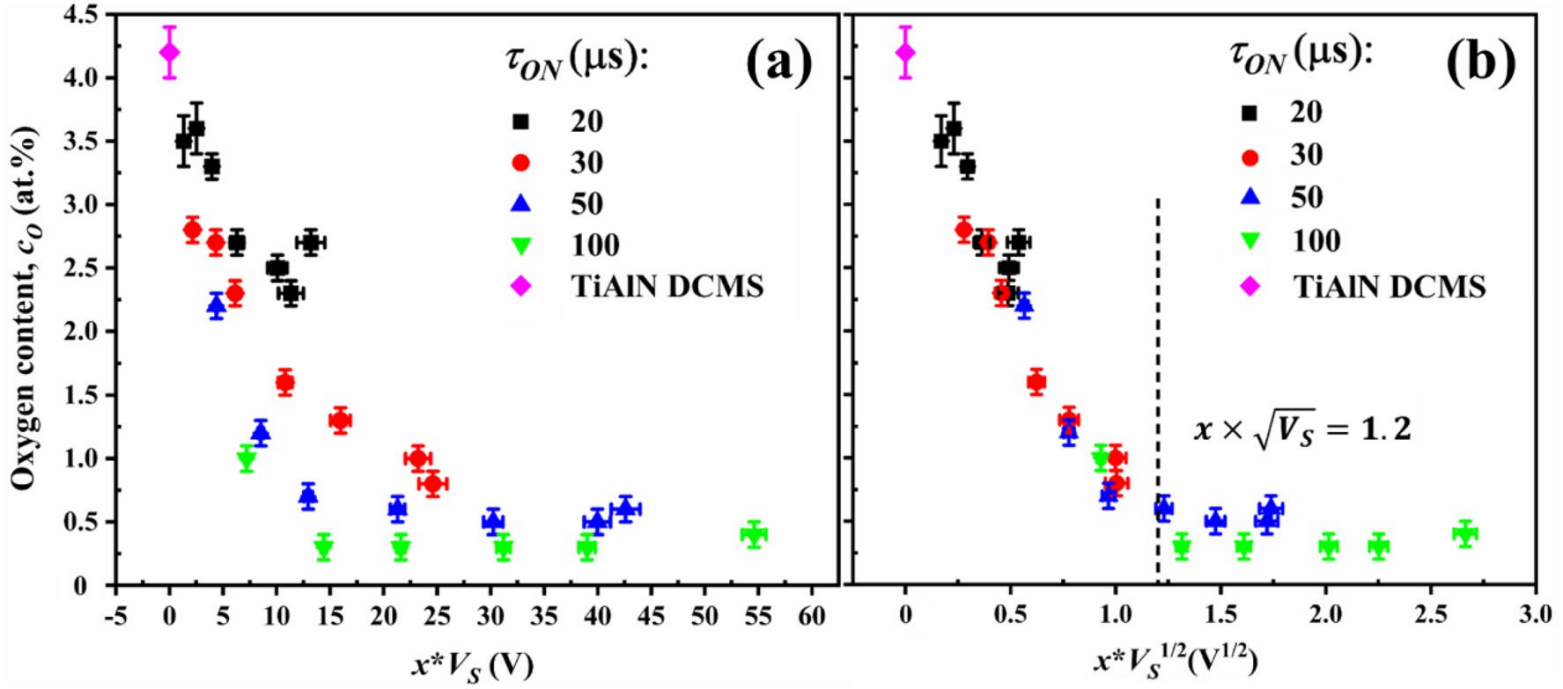
The ToF-ERDA-derived oxygen concentrations *c*_*o*_ for (Ti_1-*y*_Al_*y*_)_1-*x*_W_*x*_N films grown with the HiPIMS pulse length *τ*_*ON*_ = 20 (*x* = 0.02), 30 (*x* = 0.04), 50 (*x* = 0.07), and 100 μs (*x* = 0.12) as a function of (**a**) $x\times {V}_{s}$ and (**b**) $x\times \sqrt{{V}_{s}}$.

The approach undertaken in this contribution relies on the critical analysis of previously published results for (Ti_1-*y*_Al_*y*_)_1-*x*_W_*x*_N films with *y* ≂ 0.45. In Fig. 1 we replot the ERDA-derived oxygen content *c*_*o*_ for (Ti_1-*y*_Al_*y*_)_1-*x*_W_*x*_N films grown with *τ*_*ON*_ = 20 (*x* = 0.02), 30 (*x* = 0.04), 50 (*x* = 0.07), and 100 μs (*x* = 0.12). For each series, the amplitude of synchronized bias *V*_*s*_ was varied from 60 to 600 V in steps of 60 V. As the background system pressure during film growth is relatively low, the majority of detected oxygen is due to inward diffusion upon air exposure that scales with film porosity. Hence, *c*_*O*_ constitutes a good measure of film density, which was previously shown to be a function of both *x* and *V*_*S*_. Here, we replot *c*_*O*_ as a function of either $x\times {V}_{s}$ (Fig. 1a) or $x\times \sqrt{{V}_{s}}$ (Fig. 1b). The average energy deposited per metal atom, $\langle {E}_{d}\rangle ={E}_{i}{J}_{i}/{J}_{M}$, in which ${E}_{i}$ is the average energy of metal ions, ${E}_{i}$≃e*V*_*s*_ and ${J}_{i}/{J}_{M}$ is the ratio of the energetic metal ion flux to the flux of condensing metal atoms. If $\alpha$ denotes the ionized fraction of the W flux and $x=W/(W+Ti+Al)$, ${J}_{i}/{J}_{M}$ can be simply expressed as $\alpha x$. Thus, $\langle {E}_{d}\rangle =\alpha ex{V}_{s}$ is proportional to ${xV}_{s}$, as $\alpha$ is not expected to vary significantly due to the fact that the peak target current density during W-HiPIMS is maintained constant. In a similar way, the average momentum deposited per metal atom $\langle {p}_{d}\rangle =\alpha ex\sqrt{{V}_{s}}$. Hence, the first expression $x\times {V}_{s}$ is proportional to the energy deposited by W^+^ metal ions per all deposited metal atoms (W, Al, and Ti), while the second one $x\times \sqrt{{V}_{s}}$ is proportional to the momentum transferred by W^+^ ions per deposited metal atoms. Although the ionized fraction of the W flux from the HiPIMS cathode $\alpha$ is not known precisely, it does not impact this comparison which aims to answer the essential question—is the high mass metal ion densification process governed by energy or momentum transfer? Data shown in Fig. 1 provide strong evidence that the latter is the case. While data points plotted as a function of $x\times {V}_{s}$ show significant scatter, the obvious trend is observed in the ${c}_{O}-(x\times \sqrt{{V}_{s}})$ plot: the residual oxygen content shows a gradual decrease with increasing $x\times \sqrt{{V}_{s}}$ from values as high as 4.2 at.% for the reference Ti_0.52_Al_0.48_ N layer (no W^+^ irradiation, $x\times \sqrt{{V}_{s}}$ = 0), to 3.5 at.% with 0.1 ≲ $x\times \sqrt{{V}_{s}}$ ≲ 0.3 down to 0.3–0.5 at.% with $x\times \sqrt{{V}_{s}}$ ≂ 1.2. The latter marks the point where dense films are obtained. With further increasing $x\times \sqrt{{V}_{s}}$ , the oxygen content does not drop further, and is determined by the adsorption from the gas phase during film growth under high-vacuum conditions.

Hence, for further studies aiming at deposition of (Ti_1-*y*_Al_*y*_)_1-*x*_W_*x*_N films with *y* significantly higher than 0.45 the primary objective is to maintain $x\times \sqrt{{V}_{s}}$ ≂ 1.2 in order to ensure that dense layers are obtained. Otherwise, the analysis of Al solubility limits would not be meaningful. As can be seen from Fig. 1(b), none of the films grown with *τ*_*ON*_ = 20 or 30 μs satisfies this condition (within the investigated *V*_*s*_ range—up to 600 V). In the case of layers deposited with *τ*_*ON*_ = 50 and 100 μs, the critical *V*_*s*_ values that provide sufficient momentum transfer are 300, and 120 V, respectively. Thus, these two HiPIMS pulse length and biasing conditions are selected for further experiments.

**Figure 2:**
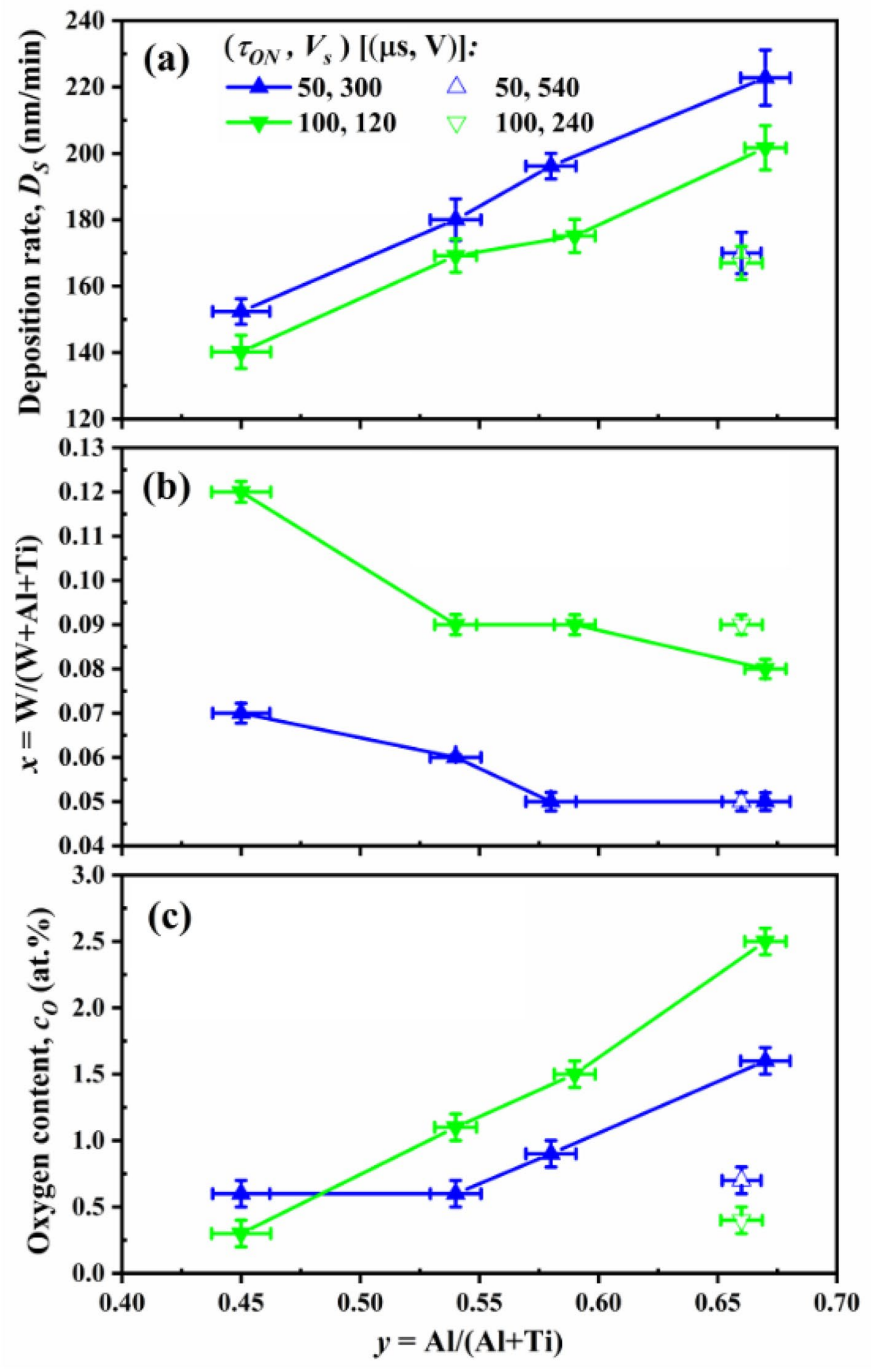
(**a**) The film growth rate *D*_*S*_ obtained from XSEM images (**b**) the ToF-ERDA-derived W content on the metal lattice, *x* = W/(W + Al + Ti) and (**c**) the ToF-ERDA-derived oxygen content *c*_*O*_ as a function of Al/(Al + Ti) ratio *y* for *τ*_*ON*_ = 50 μs and *τ*_*ON*_ = 100 μs series of (Ti_1-*y*_Al_*y*_)_1-*x*_W_*x*_N films.

Figure 2a shows the film growth rate *D*_*S*_ obtained from XSEM images as a function of Al/(Al + Ti) ratio *y* for two series of (Ti_1-*y*_Al_*y*_)_1-*x*_W_*x*_N films (*τ*_*ON*_ = 50 μs, *V*_*s*_ = 300 V and *τ*_*ON*_ = 100 μs, *V*_*s*_ = 120 V). The Al content is varied by using different sets of targets on DC positions (cf. Table 1). A remarkable increase in *D*_*S*_ with *y* is observed even though the DC power setting is kept constant at 2 × 10 and 2 × 9.5 kW for the 50 and 100 μs series, respectively. For the 50 μs series the growth rate increases from 152 nm/min with *y* = 0.45 to 223 nm/min with *y* = 0.67 (i.e., by 46.7%). Corresponding increase for the 100 μs series is from 140 to 202 nm/min. The overall *D*_*S*_ values in that case are lower due to lower DC powers used. The increase of the growth rate with increasing Al content is predominantly due to two factors. First, the fact that under the current experimental conditions (DCMS target voltage of ~ 500 V resulting in the Ar^+^ incident energy of approximately 500 eV), the sputter yield from the Al parts of the target is about twice as high as that from Ti parts^25^. Secondly, as discussed below in more detail, the film porosity increases with increasing *y* contributing to an increase of the apparent film thickness.

As a result of that, the W^+^ flux from HiPIMS source relative to the Ti and Al neutral fluxes from DCMS cathodes decreases, leading to lower W content on the metal lattice, *x* = W/(W + Al + Ti), with increasing *y*, as shown in Fig. 2b, where ERDA-derived values are plotted. For the 50 μs samples, *x* decreases from 0.07 with *y* = 45 to 0.05 with *y* = 0.67, while for films grown with longer W-HiPIMS pulses the decrease is from 0.12 to 0.08. Since the amplitude of the pulsed substrate bias is kept constant for each series, at 300 and 120 V, respectively, layers develop porosity with increasing Al content (and the TiAlN deposition rate). This is evidenced by the higher concentration of adsorbed oxygen, as depicted in Fig. 2c. Since the background system pressure during deposition is in a low 10^–6^ mbar range, the oxygen *c*_*O*_ detected by ERDA mainly comes from the air exposure, thus serving as a reliable measure of film porosity that correlates closely to mechanical properties such as hardness^21^. For (50 μs, 300 V) films, the oxygen content *c*_*O*_ increases from 0.6 with *y* = 0.45 to 1.6 at.% with *y* = 0.67. For (100 μs, 120 V) series, the *c*_*O*_ is lowest at low *y,* only 0.3 at.% and reaches 2.5 at.% with *y* = 0.67.

**Figure 3:**
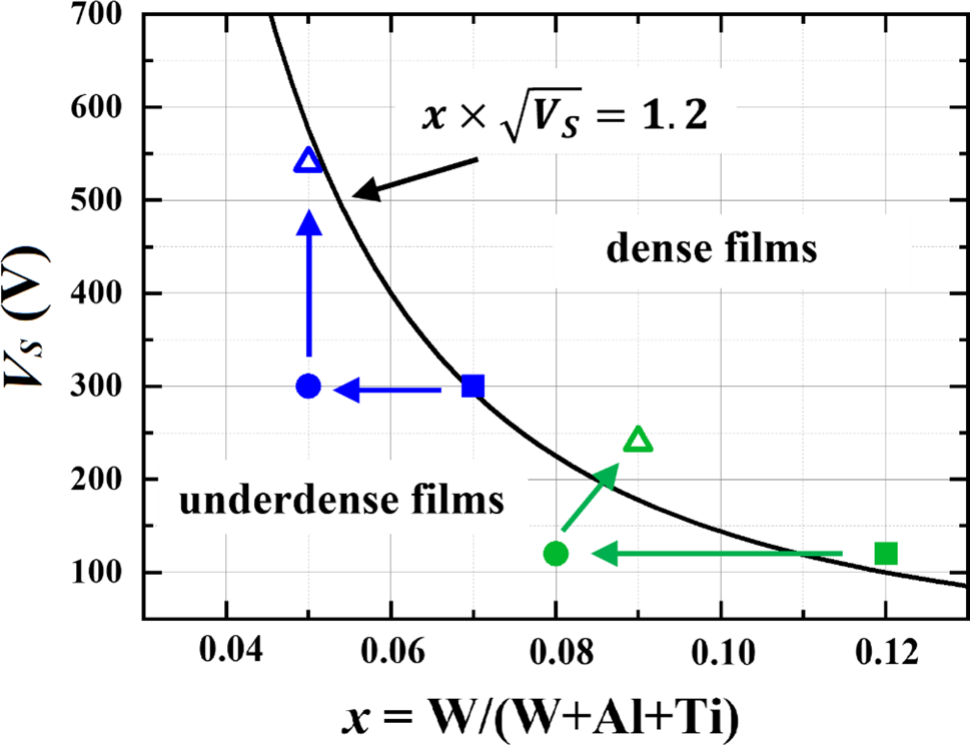
The 2D *V*_*s*_ vs. *x* map. The $x\times \sqrt{{V}_{s}}=1.2$ curve separates the regions of underdense ($x\times \sqrt{{V}_{s}}<1.2$) from dense ($x\times \sqrt{{V}_{s}}>1.2$) (Ti_1-*y*_Al_*y*_)_1-*x*_W_*x*_N films. Squares indicate layers with *y* = 0.45 (from Ref.^21^), which are dense. Circles denote porous films with the highest Al content *y* = 0.67, while triangles stand for Ti_0.32_Al_0.63_W_0.05_ N (*τ*_*ON*_ = 50 μs, *V*_*s*_ = 540 V) and Ti_0.31_Al_0.60_W_0.09_ N (*τ*_*ON*_ = 100 μs, *V*_*s*_ = 240 V) films.

The above changes are illustrated in the form of a 2D *V*_*s*_ vs. *x* map shown in Fig. 3. Squares represent films with *y* = 0.45 from Ref. 21, which are dense as evidenced by low oxygen concentrations, the lack of visible porosity in XTEM images, and high nanoindentation hardness. Circles indicate films with the highest Al content *y* = 0.67, which exhibit high O content and low hardness (~ 20 GPa) values due to apparent porosity. Also indicated in the figure is the $x\times \sqrt{{V}_{s}}$ = 1.2 curve (cf. Fig. 1), which marks the border between the underdense and dense films in the *V*_*s*_ vs. *x* parameter space. Clearly, the decrease of the W^+^ flux relative to the combined Ti and Al neutral flux caused by the use of targets with higher Al content not only dilutes the W content on the metal lattice, but more importantly shifts the growth conditions across the $x\times \sqrt{{V}_{s}}$ = 1.2 border line into the region of apparent porosity. Thus, according to Fig. 3, there are two possible paths to deposit dense (Ti_1-*y*_Al_*y*_)_1-*x*_W_*x*_N layers: (1) via increase of the substrate bias amplitude *V*_*s*_, which controls the energy and momentum of W^+^ ions (corresponding to moving along the vertical arrow in Fig. 3), or (2) by increasing the W^+^ flux relative to the metal neutral flux resulting in higher *x* (corresponding to moving in the horizontal direction in Fig. 3). The combination of both also appears as a possible way to achieve dense films. As our previous results indicate that films with higher *x* tend to develop higher compressive stresses, ^21^ we decided to choose the first option, i.e., increasing *V*_*s*_.

Hence, two additional films were grown using 50 and 100 μs W-HiPIMS pulses and the same process conditions and targets as for two films indicated with circles in Fig. 3, however with *V*_*s*_ = 540 and 240 V, respectively. These are the minimum values of substrate bias that, according to Fig. 3, ensure that films are dense (marked with triangles). The resulting compositions according to ERDA are Ti_0.32_Al_0.63_W_0.05_ N (*τ*_*ON*_ = 50 μs, *V*_*s*_ = 540 V) and Ti_0.31_Al_0.60_W_0.09_ N (*τ*_*ON*_ = 100 μs, *V*_*s*_ = 240 V). Importantly, in both cases an increase in *V*_*s*_ resulted in significantly lowered oxygen concentrations: from 1.6 to 0.6 at.%, and from 2.5 to only 0.4 at.%, respectively. This proves the correctness of our reasoning above and emphasizes the dominant role of momentum transfer for film densification by heavy ion bombardment in the absence of intentional substrate heating. For comparison, the reference Ti_0.36_Al_0.64_ N film grown by DCMS from the same type of TiAl targets but no W^+^ bombardment and otherwise the same conditions (no external heating) exhibits extreme porosity resulting in oxygen levels as high as 6.2 at.%.

**Figure 4:**
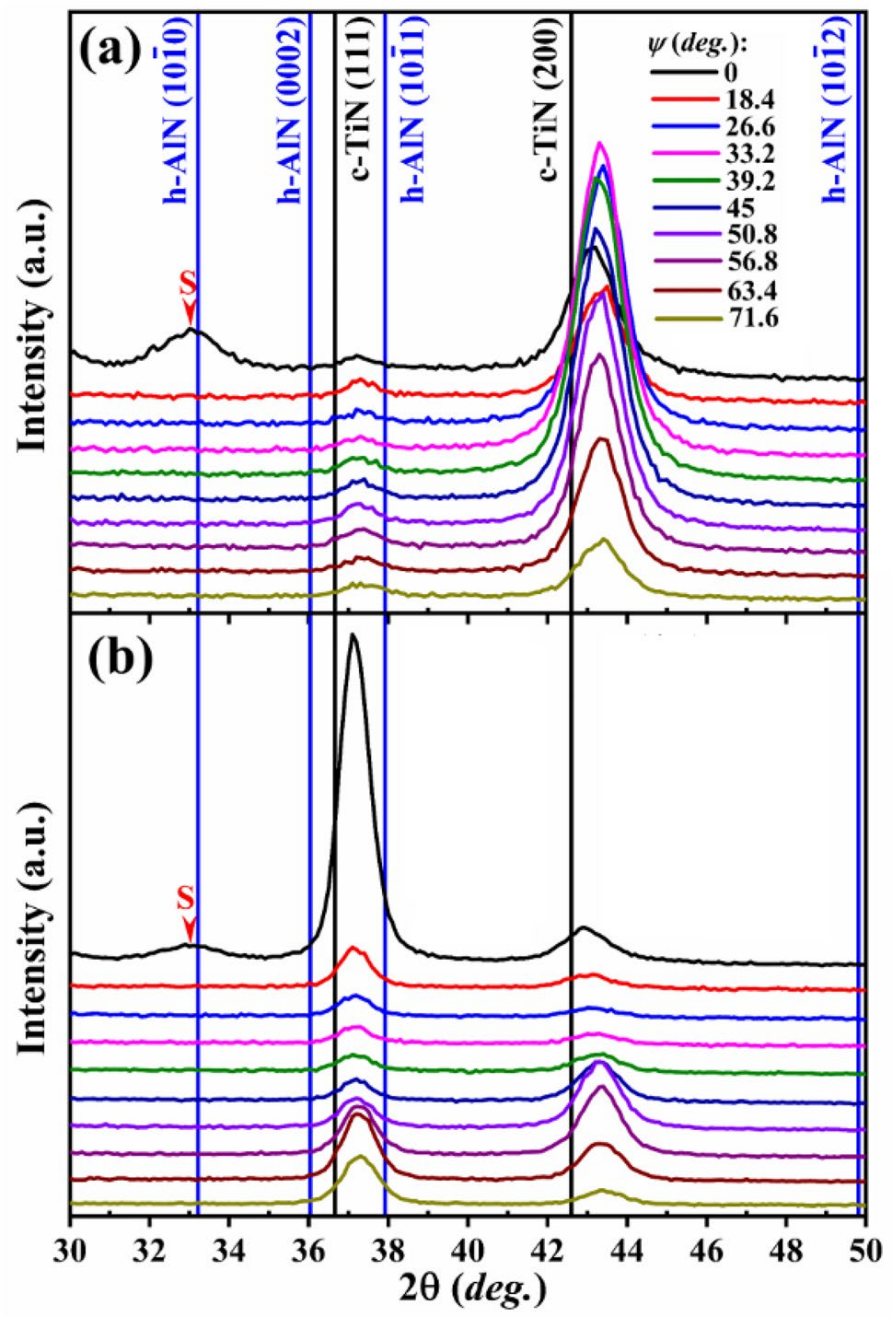
The XRD θ–2θ scans as a function of sample tilt angle ψ for the (**a**) (50 μs, 540 V), and (**b**) (100 μs, 240 V) layers with *y* = 0.66.

To investigate the phase structure the XRD θ–2θ scans as a function of sample tilt angle ψ are conducted for both (50 μs, 540 V) and (100 μs, 240 V) layers, as shown in Fig. 4. It is clearly shown in the figure that despite very high Al/(Al + Ti) ratio *y* = 0.66, films remain single phase NaCl structure, and no peaks due to hexagonal wurtzite AlN phase are observed. The Ti_0.32_Al_0.63_W_0.05_ N layer (Fig. 4a) exhibits 200 preferred orientation. Both cubic phase peaks are shifted toward higher 2θ values with respect to TiN powder diffraction patterns due to the substitution with smaller Al atoms. The relaxed lattice parameter calculated at the strain free tilt angle is 4.173 Å, which is slightly larger than 4.158 Å obtained for the reference Ti_0.36_Al_0.64_ N film with similar Al content, indicating that W is dissolved in the NaCl lattice. The Ti_0.31_Al_0.60_W_0.09_ N film grown with 100 μs pulses (Fig. 4b) exhibits 111 preferred orientation. The relaxed lattice parameter is 4.186 Å, i.e., even larger than for the Ti_0.32_Al_0.63_W_0.05_ N film, which is fully consistent with higher W concentration. For both films the change in peak positions with varying the tilt angle is small indicative of low residual stress levels.

**Figure 5:**
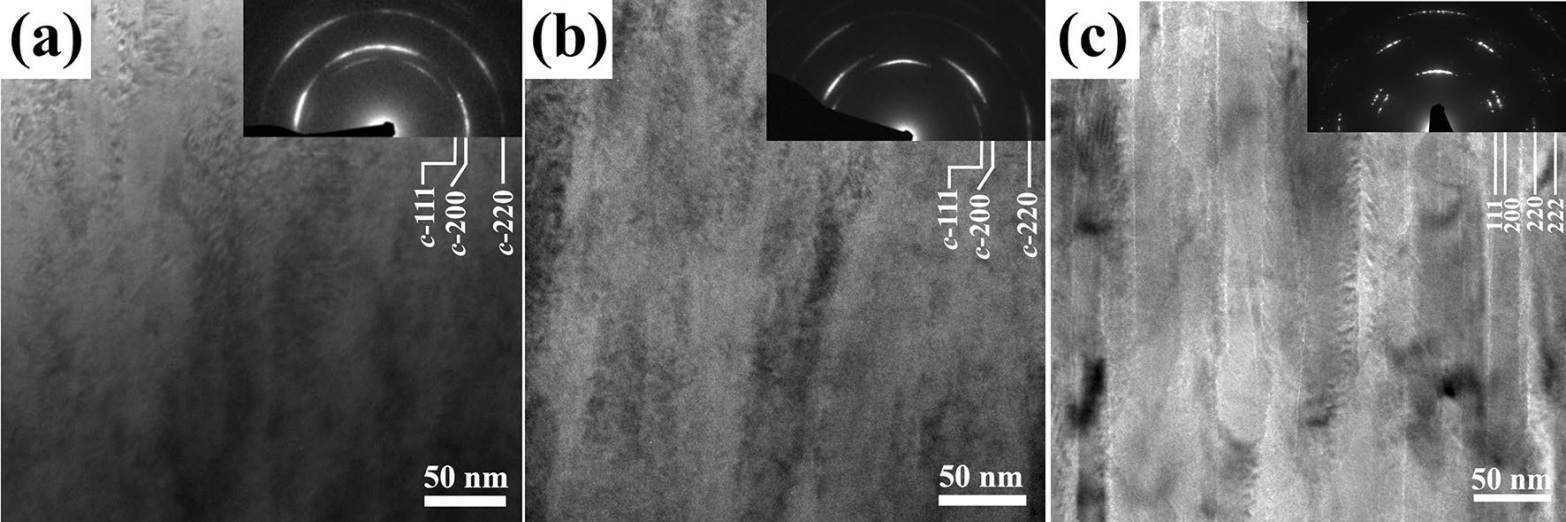
The bright field XTEM images with corresponding selected-area electron diffraction (SAED) patterns for (**a**) Ti_0.32_Al_0.63_W_0.05_ N (50 μs, 540 V), (**b**) Ti_0.31_Al_0.60_W_0.09_ N (100 μs, 240 V), and (**c**) the reference Ti_0.48_Al_0.52_ N layers. The last image is adopted from Ref.^21^.

Figure 5 shows the bright field XTEM images with their corresponding selected-area electron diffraction (SAED) patterns for (a) Ti_0.32_Al_0.63_W_0.05_ N (50 μs, 540 V), (b) Ti_0.31_Al_0.60_W_0.09_ N (100 μs, 240 V), and (c) the reference Ti_0.48_Al_0.52_ N layers. In contrast to the latter film, which exhibits pronounced porosity, both Ti_0.32_Al_0.63_W_0.05_ N and Ti_0.31_Al_0.60_W_0.09_ N layers have dense columnar structure with speckle contrast within the crystalline grains, which is typical for ion bombardment induced radiation damage in the form of strain field associated with clusters of point defects^1^. The lack of open column boundaries agrees with the interpretation that the oxygen content in these films is due to incorporation of residual gases (water and oxygen) during deposition and not adsorption upon exposure to air.

**Figure 6:**
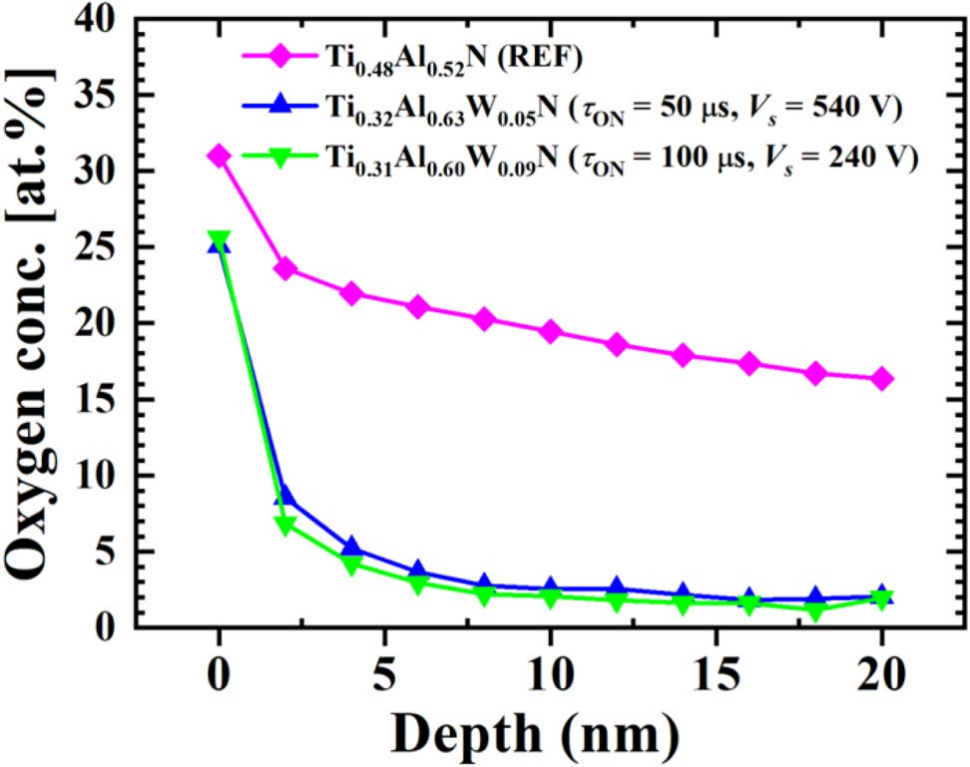
The oxygen concentration depth profiles obtained from XPS for Ti_0.32_Al_0.63_W_0.05_ N (50 μs, 540 V), Ti_0.31_Al_0.60_W_0.09_ N (100 μs, 240 V), and the reference Ti_0.48_Al_0.52_ N layer. The absolute numbers are overestimated (as compared to the results of ToF-ERDA analyses) due to the artefacts introduced by ion etching and oxygen redeposition on the surface.

Further support for this statement is provided in Fig. 6 where the oxygen concentration depth profiles obtained from XPS sputter-etching experiments are shown. In the case of the reference Ti_0.48_Al_0.52_ N film the O concentration is high and shows only a slight decay with depth, which is characteristic of a porous structure that facilitates O diffusion into the film. In contrast, O content in Ti_0.32_Al_0.63_W_0.05_ N (50 μs, 540 V) and Ti_0.31_Al_0.60_W_0.09_ N (100 μs, 240 V) samples is high only at the very surface (due to the presence of a native oxide) and drops abruptly with depth, indicative of dense films.

ERDA, XRD, and XTEM observations discussed above are fully confirmed by the results of mechanical testing. Nanoindentation hardness *H* increases with increasing substrate bias from ~ 20 GPa measured for underdense layers (circles in Fig. 3) to 27 and 30 GPa for Ti_0.32_Al_0.63_W_0.05_ N (50 μs, 540 V) and Ti_0.31_Al_0.60_W_0.09_ N (100 μs, 240 V), respectively. These numbers are in the same range as hardness of TiAlN films grown under conventional conditions, i.e., at the substrate temperature of 400–500 °C^26–31^. They are also much higher than *H* measured for the Ti_0.36_Al_0.64_ N reference film grown with no intentional heating and no W^+^ irradiation, which shows *H* = 12 GPa. High *H* values obtained for Ti_0.32_Al_0.63_W_0.05_ N and Ti_0.31_Al_0.60_W_0.09_ N films are not caused by the buildup of compressive stresses. The residual stress is low in both cases at − 0.30 ± 0.14 GPa, and − 0.71 ± 0.14 GPa, respectively.

Thus, our results provide evidence that W^+^ irradiation from HiPIMS source can be successfully used to compensate for the lack of thermally-driven adatom mobility in the absence of substrate heating during growth of (Ti_1-*y*_Al_*y*_)_1-*x*_W_*x*_N films with high Al content. Dense, single NaCl phase films with low residual stress levels are demonstrated. The key parameter that governs the growth under these conditions is the momentum transfer supplied by W^+^ irradiation per deposited neutral flux.

The supplied process energy is utilized more efficiently than in the conventional approach, as large energy losses due to heating of the entire vacuum vessel are avoided. The energy consumed by resistive heaters is reduced from 38 kWh in the conventional DCMS process used to grow reference Ti_0.36_Al_0.64_ N films to only 4 kWh in the case of Ti_0.32_Al_0.63_W_0.05_ N layers grown by W-HiPIMS/AlTi-DCMS. The extra energy cost associated with the W-HiPIMS source is 0.3 kWh, i.e., much less than the energy saved on eliminating substrate heating. Hence, the overall energy consumption is reduced by 83%. This number applies only for the specific settings used in the paper of our experiments and does not include all other ingredients such as the energy necessary for pumping down and cooling down of temperature-sensitive parts. The purpose of this estimate is not to provide the exact number, but rather to indicate how large is the saving potential of the proposed method is. Even with all other ingredients included, the saving is expected to be significant.

Independently of the significant energy savings the novel approach enables the growth of high-quality coatings on substrates such as polymers, different types of steel, or Mg alloys, that cannot be coated by conventional techniques due to high process temperatures involved. All these material classes have either low melting points or undergo phase transformation at relatively low temperatures, which prevents the use of conventional PVD techniques. Hence, the replacement of substrate heating by irradiation with heavy metal ions together with metal-ion-synchronized biasing considerably expands the range of possible applications.

## Conclusions

We have studied the W^+^ densification effects in metastable (Ti_1-*y*_Al_*y*_)_1-*x*_W_*x*_N alloys with the focus on depositing layers with *y* = Al/(Al + Ti) close to the solubility limits (*y* ~ 0.67). (Ti_1-*y*_Al_*y*_)_1-*x*_W_*x*_N films were grown by hybrid W-HiPIMS/TiAl-DCMS approach with pulsed substrate bias *V*_*S*_ synchronized to the W^+^ ion flux. To suppress the thermally-driven densification, no intentional substrate heating was used during film growth (substrate temperature was lower than 130 °C). We showed that W^+^ irradiation from HiPIMS source can be successfully used to compensate for the lack of thermally-driven adatom mobility, while avoiding precipitation of softer hexagonal wurtzite AlN phase. The key parameter that governs the growth under these conditions is the momentum transfer supplied by W^+^ irradiation per deposited neutral flux. Provided that the latter is sufficiently high, dense, single NaCl phase Ti_0.32_Al_0.63_W_0.05_ N films can be obtained. The nanoindentation hardness is comparable to that of TiAlN films grown at 400–500 °C, while the residual stresses are very low. These results indicate that the novel film growth approach, proposed by us in previous papers and aiming at significantly reduced energy consumption during PVD growth, is fully competitive against the state of the art high-temperature processing. The supplied process energy is utilized more efficiently than in the conventional approach, since large energy losses due to heating of the entire vacuum chamber are eliminated. As the consequence of that, the overall energy consumption is reduced by 83% in this particular example. The additional benefit is the expanded process window that includes temperature-sensitive substrates not possible to coat with conventional PVD.
